# Evaluation of Thyroid Dysfunction in Type 2 Diabetes Mellitus Patients and Its Association With Diabetic Complications: A Cross-Sectional Study

**DOI:** 10.7759/cureus.78871

**Published:** 2025-02-11

**Authors:** Prasanna Ratilal Patel, Aishika Maitra, Arya Ashok, JoseKutty Jose, Yashwanth Ragav, Nikitha Novalene Paul

**Affiliations:** 1 Department of General Medicine, Gujarat Medical Education and Research Society (GMERS) Medical College and Hospital, Valsad, IND; 2 Department of General Medicine, Institute of Post-Graduate Medical Education and Research and Seth Sukhlal Karnani Memorial Hospital, Kolkata, IND; 3 Department of Respiratory Medicine, Al-Azhar Medical College and Super Speciality Hospital, Thodupuzha, IND; 4 Department of General Medicine, Government Medical College Kottayam, Kottayam, IND; 5 Department of General Medicine, Thoothukudi Government Medical College Hospital, Thoothukudi, IND; 6 Department of General Medicine, Government Medical College Anantapur, Anantapur, IND

**Keywords:** diabetic nephropathy, diabetic retinopathy, hypothyroidism, peripheral arterial disease, thyroid dysfunction, type 2 diabetes mellitus

## Abstract

Background

Type 2 diabetes mellitus (T2DM) is a widespread metabolic disorder that contributes significantly to morbidity and mortality due to its associated complications. Among these, thyroid dysfunction is one of the most common and may play a role in the progression of diabetic complications. This study explores the relationship between thyroid dysfunction and both microvascular and macrovascular complications in individuals with T2DM.

Methods

This cross-sectional observational study was conducted among 200 patients with T2DM, aged 30-70 years, at the Department of General Medicine, Gujarat Medical Education and Research Society (GMERS) Medical College and Hospital, Valsad, India. Participants were screened for thyroid dysfunction and diabetic complications, including retinopathy, nephropathy, neuropathy, and peripheral arterial disease (PAD). Blood tests were performed to measure thyroid hormone levels (thyroid-stimulating hormone, free triiodothyronine, and free thyroxine) and diabetes-related parameters such as glycated hemoglobin (HbA1c). Data were analyzed using chi-square tests and multivariate regression analysis to evaluate the correlation between thyroid dysfunction and the development of diabetic complications.

Results

The prevalence of thyroid dysfunction in the study population was 26.5%, with hypothyroidism being more common (84.9%) than hyperthyroidism (15.1%). Diabetic complications were significantly more prevalent among individuals with hypothyroidism compared to those with normal thyroid function. Hypothyroidism was independently associated with increased odds of diabetic retinopathy (OR = 7.72), diabetic nephropathy (OR = 4.87), neuropathy (OR = 3.10), and PAD (OR = 8.12). In contrast, hyperthyroidism showed no significant association with diabetic complications.

Conclusions

Hypothyroidism in individuals with T2DM is significantly associated with a higher risk of both microvascular and macrovascular complications. These findings highlight the importance of incorporating thyroid function assessment into routine diabetic care, as optimizing thyroid hormone levels may help mitigate the risk of these complications.

## Introduction

Type 2 diabetes mellitus (T2DM) is a chronic metabolic disorder characterized by insulin resistance, leading to significant health complications and increased mortality. According to the International Diabetes Federation, approximately 463 million people worldwide have T2DM, representing 9.3% of the global adult population. This number is projected to rise to 700 million by 2045 [1]. The prevalence of T2DM varies across regions, with the highest rates observed in Southeast Asia, the Middle East, and North Africa. India faces a growing burden of T2DM, with an estimated 77 million affected individuals, translating to a prevalence of 8-10% among adults. Projections indicate that by 2045, the number of cases in India could surpass 100 million [1].

Thyroid dysfunction has emerged as a significant comorbidity in T2DM, influencing metabolic regulation and contributing to disease-related morbidity [2]. Globally, the pooled prevalence of thyroid dysfunction in individuals with T2DM is estimated at 20.24% [3]. The specific prevalence of subclinical hypothyroidism, hypothyroidism, subclinical hyperthyroidism, and hyperthyroidism in T2DM is reported to be 11.87% [3]. In India, the prevalence of thyroid dysfunction among T2DM patients is approximately 16.2%, although it varies across different regions [4,5].

Metabolic dysregulation and insulin resistance in T2DM promote thyroid growth, increasing the likelihood of nodular formations and thyroid dysfunction. Additionally, chronic inflammation and oxidative stress disrupt the hypothalamic-pituitary-thyroid axis, often resulting in hypothyroidism or subclinical thyroid dysfunction [6]. Among T2DM patients, subclinical hypothyroidism is the most common form, although overt hypothyroidism and hyperthyroidism are also observed [6].

Previous studies have established a significant relationship between thyroid hormone levels and the development of microvascular complications in T2DM. An inverse relationship has been suggested between free triiodothyronine (FT3) and free thyroxine (FT4) levels and the occurrence of microvascular complications in T2DM, whereas thyroid-stimulating hormone (TSH) levels have shown a positive correlation with these complications [7]. Additional studies indicate that low-to-normal FT3 and FT4 levels, alongside elevated TSH, are linked to an increased risk of diabetic nephropathy, particularly in cases of macroalbuminuria [8]. Furthermore, low FT4 and high TSH levels have been associated with the progression of diabetic retinopathy. Evidence suggests that FT3, FT4, and TSH levels are correlated with insulin resistance and chronic diabetic complications, including carotid atherosclerosis and diabetic peripheral neuropathy (DPN) [9].

Thyroid dysfunction and T2DM share a complex, bidirectional pathophysiological relationship, where thyroid disorders influence glucose metabolism, and diabetes affects thyroid hormone regulation. Hypothyroidism reduces insulin sensitivity, worsening glycemic control, while hyperthyroidism increases insulin resistance and hepatic glucose output. The impact of thyroid disorders on T2DM varies - hypothyroidism is associated with dyslipidemia and weight gain, whereas hyperthyroidism exacerbates hyperglycemia. Although less studied, thyroid malignancies may also affect metabolism and insulin resistance through systemic inflammation and hormonal imbalances [10].

Against this backdrop, the present study aims to evaluate thyroid dysfunction in T2DM patients and its association with microvascular and macrovascular complications.

## Materials and methods

This cross-sectional observational study was conducted among T2DM patients aged 30-70 years attending the outpatient and inpatient departments of general medicine. The study took place at the Department of General Medicine, Gujarat Medical Education and Research Society (GMERS) Medical College and Hospital, Valsad, India, over a one-year period from September 2023 to September 2024. Ethical approval was obtained from the institutional ethics committee (approval number GMERS/MCV/IHEC/76/23), and all participants provided written informed consent before enrollment. A total of 200 T2DM patients were included in the study, based on a power analysis with a 95% confidence level, 80% power, and a 5% margin of error.

Inclusion criteria

Individuals aged 18 years or older with a prior diagnosis of T2DM, as defined by the American Diabetes Association [11], and exhibiting normal thyroid function were included in the study.

Exclusion criteria

Patients with a known history of thyroid disorders, including hypothyroidism, hyperthyroidism, or autoimmune thyroiditis, as well as those receiving thyroid hormone replacement therapy or with a history of thyroid surgery, were excluded from the study. Additionally, patients with acute diabetic complications or comorbidities that could affect thyroid function (e.g., malignancy and severe liver disease) were not included.

Study procedure

The demographic data of each participant, including age, sex, and anthropometric parameters, were recorded. Venous blood samples, approximately 5 mL, were collected from each patient in the morning following an overnight fasting period. These samples were analyzed for blood glucose levels, glycated hemoglobin (HbA1c), FT3, FT4, and TSH. Blood glucose levels were measured using the enzymatic method, while HbA1c concentrations were determined using high-pressure liquid chromatography. Serum levels of T3, T4, and TSH were quantified using the chemiluminescence immunoassay (CLIA) technique.

Thyroid dysfunction was defined based on deviations from established reference ranges. Overt hypothyroidism was characterized by TSH levels greater than 5.5 μIU/mL, free T4 levels below 0.8 ng/dL, and/or free T3 levels below 1.4 pg/mL. Overt hyperthyroidism was diagnosed when TSH levels were below 0.5 μIU/mL, with free T4 levels exceeding 1.5 ng/dL and/or free T3 levels above 4.2 pg/mL. Subclinical hypothyroidism was defined by TSH levels greater than 5.5 μIU/mL while maintaining normal free T3 and free T4 levels. Subclinical hyperthyroidism was characterized by TSH levels below 0.5 μIU/mL with normal free T3 and free T4 levels.

Diabetic complications were assessed based on standard diagnostic criteria. Diabetic retinopathy was diagnosed using fundoscopy or retinal photography. Diabetic nephropathy was evaluated by measuring the urinary albumin-to-creatinine ratio and serum creatinine levels. The severity of nephropathy was classified according to albumin excretion rates, with values below 30 mg/g considered normal, between 30 and 300 mg/g indicating microalbuminuria, and levels above 300 mg/g classified as macroalbuminuria. Diabetic neuropathy was diagnosed using the monofilament test and the assessment of vibration perception threshold. Peripheral arterial disease (PAD) was identified using the ankle-brachial index, with a value of less than 0.9 indicating the presence of PAD.

Statistical analysis

Continuous variables were expressed as mean ± SD, while categorical variables were presented as frequencies (%). The association between thyroid dysfunction and microvascular and macrovascular complications was assessed using the chi-square test. Multivariate regression analysis was conducted to evaluate the independent effects of thyroid dysfunction on diabetic complications. Data analysis was performed using IBM SPSS Statistics for Windows, Version 24.0 (Released 2016; IBM Corp., Armonk, NY, USA).

## Results

The demographics and clinical characteristics of the study population are summarized in Table 1. The mean age of the patients was 55.6 ± 8.4 years, with a male predominance observed in 125 (62.5%) cases. A majority of patients, 144 (72%), had a BMI greater than 25 kg/m². The mean HbA1c level was 8.13 ± 1.65, and 172 (86%) of the patients had an HbA1c level above 7%. The overall prevalence of thyroid dysfunction among T2DM patients in this study was 53 (26.5%). Of these, 45 (84.9%) had hypothyroidism, while eight (15.1%) had hyperthyroidism.

**Table 1:** Demographics and clinical characteristics of the study participants

Variables	No. of patients (n = 200), n (%)
Age (years) (mean ± SD)	55.6 ± 8.4
Gender	
Male	125 (62.5%)
Female	75 (37.5%)
Duration of diabetes (years) (mean ± SD)	8.53 ± 4.74
BMI (kg/m²) (mean ± SD)	27.42 ± 4.63
BMI >25 kg/m², n (%)	144 (72%)
HbA1c (%) (mean ± SD)	8.13 ± 1.65
HbA1c >7%, n (%)	172 (86%)
Thyroid function, n (%)	
Normal	147 (73.5%)
Thyroid dysfunction	53 (26.5%)
Hypothyroidism (subclinical + overt)	45 (84.9%)
Hyperthyroidism	8 (15.1%)
Treatment, n (%)	
Oral hypoglycemic agents	156 (78%)
Insulin	12 (6%)
Insulin + oral hypoglycemic agents	32 (16%)

The association between diabetes duration and thyroid dysfunction is presented in Table 2. The prevalence of hypothyroidism (18 patients, 40%) and hyperthyroidism (four patients, 50%) was highest among individuals with a diabetes duration of 6-10 years. This association was statistically significant (p = 0.001).

**Table 2:** Association between duration of diabetes and thyroid dysfunction The data is presented as n (%). The asterisk (^*^) indicates statistical significance (p < 0.05) based on the chi-square test.

Duration of diabetes	Normal thyroid function (n = 144)	Hypothyroidism (n = 45)	Hyperthyroidism (n = 8)	Chi square test (X²); p value
1-5 years	40 (27.8%)	10 (22.2%)	2 (25%)	X² = 14.23; p = 0.001^*^
6-10 years	55 (38.2%)	18 (40%)	4 (50%)	
11-15 years	30 (20.8%)	10 (22.2%)	1 (12.5%)	
>15 years	19 (13.2%)	7 (15.6%)	1 (12.5%)	

The association between thyroid dysfunction and diabetic complications is summarized in Table 3. Among the 53 patients with thyroid dysfunction, 43 had diabetic complications. Specifically, 38 out of 45 patients with hypothyroidism and five out of eight patients with hyperthyroidism experienced diabetic complications.

**Table 3:** Association between thyroid dysfunction and diabetic complications The data is presented as n (%). The asterisk (^*^) indicates statistical significance (p < 0.05) based on the chi-square test. PAD, peripheral arterial disease

Diabetic complications	Hypothyroidism (n = 45)	Hyperthyroidism (n = 8)	Normal thyroid function (n = 144)	Chi-square value (χ²)	p-value
Diabetic retinopathy	14 (31.1%)	2 (25%)	18 (12.5%)	15.85	0.001^*^
Diabetic nephropathy	10 (22.2%)	1 (12.5%)	12 (8.3%)	11.24	0.001^*^
Diabetic neuropathy	9 (20%)	1 (12.5%)	16 (11.1%)	8.13	0.003^*^
PAD	5 (11.1%)	1 (12.5%)	8 (5.5%)	6.87	0.02^*^

The prevalence of diabetic retinopathy (14; 31.1%), diabetic nephropathy (10; 22.2%), and diabetic neuropathy (nine; 20%) was significantly higher in patients with hypothyroidism compared to those with hyperthyroidism and normal thyroid function. However, the incidence of PAD was higher in hyperthyroid patients (one; 12.5%) compared to hypothyroid patients (five; 11.1%), and this difference was statistically significant (p = 0.02).

The association between thyroid dysfunction and diabetic complications, as assessed by multivariate analysis, is presented in Table 4. Hypothyroidism was significantly associated with a higher risk of diabetic retinopathy (OR = 7.72), diabetic nephropathy (OR = 4.87), and PAD (OR = 8.12), indicating a strong correlation with these complications. In contrast, hyperthyroidism did not show a significant association with diabetic complications (p > 0.05).

**Table 4:** Association between thyroid dysfunction and diabetic complications using multivariate analysis The asterisk (^*^) denotes statistical significance (p < 0.05); NS indicates nonsignificant results. PAD, peripheral arterial disease

Complication	Hypothyroidism (OR)	95% CI	p-value	Hyperthyroidism (OR)	95% CI	p-value
Diabetic retinopathy	7.72	1.02-10.54	0.001^*^	1.15	0.35-3.78	0.35^NS^
Diabetic nephropathy	4.87	1.11-9.93	0.001^*^	1.42	0.51-3.91	0.30^NS^
Diabetic neuropathy	3.1	0.65-5.34	0.01^*^	0.78	0.33-1.86	0.74^NS^
PAD	8.12	0.98-11.34	0.002^*^	0.95	0.30-3.07	0.82^NS^

## Discussion

This study investigated the association between thyroid dysfunction and the development of microvascular and macrovascular complications in individuals with T2DM. The findings reveal a significant correlation between thyroid dysfunction, particularly hypothyroidism, and an increased risk of diabetic complications, including diabetic retinopathy, diabetic nephropathy, diabetic neuropathy, and PAD. These results contribute to the growing body of evidence linking thyroid dysfunction to the progression of diabetes-related complications.

In this cohort, the prevalence of thyroid dysfunction was 26.5%, with hypothyroidism accounting for the majority of cases (84.9%) and hyperthyroidism observed in 15.1% of affected individuals. A study by Jalal et al. reported a thyroid dysfunction prevalence of 16% among T2DM patients [12], while Asuti et al. estimated it at 23.6%, with 67.79% of cases classified as subclinical hypothyroidism and 27.11% as overt hypothyroidism [6].

A significant association was also observed between the duration of diabetes and thyroid dysfunction, with the highest prevalence of both hypothyroidism and hyperthyroidism occurring in patients with diabetes for 6-10 years. This suggests that prolonged exposure to diabetic conditions may increase the likelihood of developing thyroid abnormalities. The significant association (p = 0.001) indicates that thyroid dysfunction is more prevalent in individuals with a longer duration of diabetes, possibly due to complex interactions between thyroid hormones and metabolic control. In a study by Nair et al., thyroid dysfunction was significantly more common in patients with a diabetes duration of more than two years compared to those with a shorter duration (70.8% vs. 60.1%; p = 0.03) [13].

Our findings demonstrate a strong correlation between hypothyroidism and microvascular complications, particularly diabetic retinopathy, nephropathy, and neuropathy. The ORs for diabetic retinopathy, nephropathy, and PAD in hypothyroid patients were 7.72, 4.87, and 8.12, respectively, indicating a significantly higher risk of these complications. These results align with previous studies that have reported a higher prevalence of diabetic retinopathy and nephropathy in individuals with hypothyroidism.

A cross-sectional study found that, after adjusting for confounding factors, TSH levels were independently associated with DPN in T2DM patients (OR = 1.365, p < 0.01) [14]. Furthermore, Zhu and Yang identified a significant correlation between FT3 levels and altered nerve conduction in T2DM patients with euthyroid status. One key contributor to diabetic nephropathy is uncontrolled hyperglycemia, which increases the formation of advanced glycation end-products, leading to inflammation and kidney fibrosis [15]. Thyroid hormones, particularly T3, play a crucial role in regulating glucose metabolism, insulin sensitivity, and lipid metabolism. In hypothyroidism, reduced T3 levels contribute to insulin resistance, exacerbating hyperglycemia and accelerating the pathogenesis of diabetic nephropathy [16].

A study by Warris et al. found a significantly higher prevalence of diabetic retinopathy in T2DM patients with subclinical hypothyroidism compared to controls (43.3% vs. 16%; p = 0.001) [17]. Similarly, a meta-analysis by Wu et al. reported that subclinical hypothyroidism increases the risk of diabetic retinopathy by 2.13 times in T2DM patients [18]. Another study demonstrated that patients in the highest quartile of FT3 levels had a lower risk of diabetic retinopathy progression (OR = 0.368) [19].

In a study by Furukawa et al., multivariate analysis revealed a significant association between diabetic nephropathy and subclinical hypothyroidism (p = 0.03, OR = 3.51) [20]. Additionally, Nsr-Allah et al. reported that subclinical hypothyroidism increased the risk of diabetic nephropathy by 1.86 times in T2DM patients (p = 0.03) [21]. Previous research has highlighted that subclinical hypothyroidism is a risk factor for albuminuria in T2DM patients, potentially due to its impact on renal physiology, including alterations in renal blood flow, glomerular filtration rate, tubular transport mechanisms, and overall kidney structure, leading to increased protein permeability across the glomeruli [22].

A systematic review and meta-analysis by Han et al. identified subclinical hypothyroidism as a significant risk factor for PAD in T2DM patients (OR = 1.85) [23]. A key mechanism linking hypothyroidism to PAD is endothelial dysfunction, which is a hallmark of both PAD and atherosclerosis. Thyroid hormones, particularly T3, regulate endothelial function by promoting nitric oxide (NO) production, which facilitates vasodilation and vascular health. In hypothyroidism, reduced NO production leads to vasoconstriction and diminished blood flow, promoting atherosclerosis and plaque formation in peripheral arteries, which are key features of PAD [24].

This study also found that hyperthyroidism had a lesser impact on diabetic complications compared to hypothyroidism. Similarly, Mehalingam et al. reported no significant association between hyperthyroidism and diabetic complications such as retinopathy and neuropathy [25].

Limitations

Despite these significant findings, this study has several limitations. First, it was a single-center study with a relatively small sample size. Additionally, the study did not specifically account for subclinical thyroid dysfunction or autoimmune thyroid disease, both of which could further clarify the mechanisms through which thyroid hormones influence diabetic complications.

## Conclusions

This study emphasizes the strong association between thyroid dysfunction, particularly hypothyroidism, and an increased risk of microvascular complications in T2DM. Hypothyroidism was identified as an independent predictor of diabetic retinopathy, nephropathy, neuropathy, and PAD, a macrovascular complication, whereas hyperthyroidism had a less significant impact. These findings highlight the importance of routine thyroid function screening in diabetic patients and suggest that optimizing thyroid hormone levels may help mitigate diabetes-related complications. Future research should investigate the potential benefits of thyroid hormone therapy in managing these complications.
